# Sexual-orientation differences in risk of health-related impaired ability to work and to remain in the paid workforce: a prospective population-based twin study

**DOI:** 10.1186/s12889-023-15384-6

**Published:** 2023-03-08

**Authors:** Richard Bränström, Jurgita Narusyte, Pia Svedberg

**Affiliations:** 1 Division of Psychology, Department of Clinical Neuroscience, Karolinska Institutet, Nobels väg 9, 171 77 Stockholm, Sweden; 2 Division of Insurance Medicine, Department of Clinical Neuroscience, Karolinska Institutet, Stockholm, Sweden

**Keywords:** Sick leave, Disability pension, Sexual minorities, Work ability

## Abstract

**Background:**

Studies consistently show an increased risk of poor health among sexual minorities (i.e., those identifying as lesbian, gay, bisexual [LGB] or other non-heterosexuals individuals), as compared to those identifying as heterosexual. It is largely unknown whether the increased risk of mental and physical health problems among sexual minorities is also reflected in an increased risk of health-related impaired ability to work, in terms of sickness absence (SA) and disability pension (DP), or successfully remain in the paid workforce. This study made use of a large sample of Swedish twins with self-reported information about sexual behavior in young adulthood to examine sexual orientation difference in SA and DP during a 12-year follow-up period.

**Method:**

Data from the Swedish Twin project of Disability pension and Sickness absence (STODS), including Swedish twins born 1959–1985 was used (N = 17,539; n = 1,238 sexual minority). Self-report survey data on sexual behavior was linked to information about SA and DP benefits from the MicroData for Analysis of the Social Insurance database (MiDAS), the National Social Insurance Agency. Sexual orientation differences in SA and DP between 2006 and 2018 was analyzed, as well as, the influence of sociodemographic, social stress exposure (i.e., victimization, discrimination), mental health treatment, and family confounding on these differences.

**Results:**

Compared to heterosexuals, sexual minorities were more likely to having experienced SA and having been granted DP. The odds were highest for DP, where sexual minorities were 58% more likely to having been granted DP compared to heterosexuals. The higher odds for SA due to any diagnosis could largely be explained by sociodemographic factors. The higher odds of SA due to mental diagnosis could partially be explained by increased risk of being exposed to discrimination and victimization, and partially by having received treatment with antidepressant medication. The higher odds of being granted DP could also partially be explain by increased risk of being exposed to social stress and treatment with antidepressant medication.

**Conclusion:**

To our knowledge, this is the first study to report on sexual orientation differences in risk of SA and DP in a population-based sample. We found higher period prevalence of both SA and DP among sexual minorities as compared to heterosexuals. The higher odds of SA and DP could partially or fully be explained by sexual orientation differences in sociodemographic factors, exposure to social stress, and antidepressant treatment for depression. Future studies can extend these findings by continuing to investigate risk factors for SA and DP among sexual minorities and how such factors can be reduced.

## Introduction

An increasing number of studies provide evidence for substantial disparities in prevalence of mental health problems based on sexual orientation [1–7]. These studies consistently show an increased risk of mental health problems among sexual minorities (i.e., those identifying as lesbian, gay, bisexual [LGB] or other non-heterosexuals individuals), as compared to those identifying as heterosexual, with the most common mental health outcomes being: depression, anxiety, and substance use disorders [4, 8–11]. In addition to differences in mental health, several recent studies have demonstrated an increased prevalence of certain physical health outcomes among sexual minority individuals relative to heterosexuals, e.g., cardiovascular disease, asthma, diabetes, and other chronic conditions [12–14]. However, it is largely unknown whether the increased risk of mental and physical health problems among sexual minorities is also reflected in an increased risk of health-related impaired ability to work, in terms of sickness absence (SA) and disability pension (DP), or successfully remain in the paid workforce.

The increased risk of poor health among sexual minority individuals have largely been explained by exposure to sexual-orientation specific stress, also called *minority stress*, related to membership in a socially disadvantaged and stigmatized group [15]. According to minority stress theory, sexual minority people are more likely to experience stigma-based psychosocial stressors (e.g., discrimination, violence, threats, and hate crimes) that are unique and linked to their sexual identity. Exposure to these stressors across the life course is believed to compound the burden of general life stress and generate higher rates of stress-related health concerns [16]. Although these stigma-based psychosocial stressors are the most frequently explored determinants of sexual minority health, some studies have tried to assess the impact of genetic and shared environmental factors using data on siblings and twins, in predicting the increased risk of poor health among sexual minorities [17–19]. These studies give some support for a genetic covariation between mental health and sexual behavior [20], but also give support for the minority stress hypothesis and the importance of sexual minorities’ exposure to environmental stressors as contributors to risk of mental health problems [17].

Although the increased risk of poor health among sexual minorities as compared to heterosexuals is well established, whether this risk also entail a higher risk of impaired ability to work or successfully remain in the paid workforce is yet largely unknown [21]. It is also unknown if the previously identified psychosocial determinants of health disparities based on sexual orientation described above, can help explain potential differences in SA and DP based on sexual orientation. Since previous studies have demonstrated an increased risks of common mental disorders among sexual minority individuals [1, 2, 22] and mental disorders are the most common reason for work disability [23], there are reasons to assume that SA and DP are more prevalent among sexual minority people compared to heterosexuals. However, there is currently insufficient knowledge describing the prevalence and determinants of sexual orientation disparities in work disability.

This study made use of a large sample of Swedish twins with self-reported information about sexual behavior in young adulthood and links to follow-up information about reduced work ability, in terms of SA and DP, from comprehensive national registries between 2006 and 2018. The large sample size and unique data structure permitted us to pursue the following research questions: (a) Are sexual minority individuals at a higher risk of reduced work ability in terms of SA or DP due to mental and somatic disorders as compared to heterosexual individuals? (b) Can socioeconomic status (i.e., level of education) or greater exposure to commonly proposed predictors of sexual orientation disparities in health, such as, psychosocial stressors (i.e., discrimination and hate crime), explain or partially explain any potential elevated risk of reduced work ability in terms of SA and DP among sexual minorities as compared to heterosexuals? (c) Can an elevated risk of SA or DP among sexual minorities be explained by a higher prevalence of common mental health problems, such as depression, among sexual minorities as compared to heterosexuals? And (d) Can any potential sexual orientation differences in SA or DP be partially or fully explained by familial (i.e., genetic, and shared environmental) factors?

## Method

Data from the study called: Swedish Twin project of Disability pension and Sickness absence (STODS) were used. STODS include survey data from the Swedish Twin Adults: Genes and Environment (STAGE). In STAGE, all twins in the Swedish Twin Registry (STR) [17, 24, 25] born 1959–1985, where both twins in a pair were alive and living in the country, were invited to participate in a large web-based survey in 2005–2006 including items on sexual behavior. A total of 25,381 (60%) of those invited participated [25]. In STODS, self-report survey data was linked to information about SA and DP benefits from the MicroData for Analysis of the Social Insurance database (MiDAS), the National Social Insurance Agency. Information on sex (women/men), age (continuous variable), and zygosity (monozygotic, dizygotic) were obtained from the STR. To successfully categorize participants into groups by sexual orientation, responses to the questions on sexual behaviour was a requirement for inclusion. On the questions about sexual behaviour, there were over 7,000 individuals for whom the data were missing or who did not want to respond, or who did not report any same-sex or opposite-sex experiences. In the current study, the final sample included 17,539 individuals with 6,540 monozygotic (MZ) twins, 5,180 dizygotic (DZ) same-sexed twins, and 5,367 opposite-sexed twins. Information about zygosity was missing for 452 participants.

### Exposure variable

Sexual orientation was assessed using survey responses concerning same-sex sexual behavior and was defined using responses to the questions: “How many people with opposite sex have you been sexually together with?” and “How many people with same sex have you been sexually together with?” Respondents were categorized into a dichotomous variable as either a sexual minority individual (i.e., with values 1 = At least one same-sex sexual partner) or as a heterosexual individual (i.e., 0 = No same-sex sexual partner). Individuals with no response to the questions or with response “0” to both questions were excluded from further analysis.

### Outcome variables

Our main outcome variables were: (a) reimbursement due to at least one SA spell (> 14 days) during the follow-up period; or (b) reimbursement due to DP during the follow-up period. We also used the following outcome variables: (c) reimbursement due to at least one SA spell related to any mental diagnoses; or (d) reimbursement due to DP during the follow-up period related to any mental diagnoses. All individuals in Sweden with an income from work or social benefits, older than 15 years, who have a reduced ability to work due to disease or injury, can be granted SA benefits from the Social Insurance Agency while all individuals in Sweden aged 19–64 years can be granted DP if they permanently lose their ability to work due to disease or injury [26]. The follow-up period was from the date of responding to the STAGE survey (2005–2006) until the 31st of December 2018.

### Covariates/Confounders

Covariates including: age, sex, educational level (assessed based on total number of years of education categorized into three groups: 0 = 1–9 years, 1 = 10–12 years, 2 = > 12 years), relationship status (0 = single/widow, 1 = married/cohabiting), and perceived discrimination (assessed with the survey item: “Have you ever been discriminated against in a way that was highly distressing or disturbing because of your race, ethnic group, gender, sexual orientation, or religion?”: 0 = No, 1 = Yes) and/or hate crime (assessed with the survey item: “Have you ever been the victim of a hate crime?”: 0 = No, 1 = Yes) were measured at the baseline, that is, at the time of STAGE interview. Information on annual disposable income in 2016 were retrieved from the longitudinal integrated database for health insurance and labor market studies (LISA), Statistics Sweden [27]. Information on previous prescription of antidepressants (ATC: N06A) during the follow-up period 2006–2018 was retrieved from the Swedish Prescribed Drug Registry held at the National Board of Health and Welfare. Use of antidepressant was added as a covariate in the analyses as an indicator of common mental health disorders since the risk of depression has consistently been found elevated in sexual minority populations [11].

### Statistical analysis

First, descriptive statistics of all variables were calculated in the whole study population. In the analyses of sexual orientation differences in SA, all individuals with an ongoing SA spell at the time of the survey assessment were excluded (*n* = 199). Second, logistic regression was applied to the whole sample to calculate odds ratios (OR) with 95% Confidence Intervals (CI) for the associations between the exposure and the outcome variables. Twin dependency within pairs was considered by using Generalized Estimation Equations (GEE). The analyses were also adjusted for the covariates that were entered in separate steps. Third, potential familial confounding was tested in a subsample of same-sex MZ and DZ twin pairs discordant for the exposure (i.e., so called cotwin control analysis), that is, twin pairs where one twin in a pair reported on same-sex sexual behavior and the other not. ORs were calculated using conditional logistic regression and compared to the estimates of the whole sample. Attenuated estimates in a subsample of exposure discordant twin pairs would suggest that familial factors (genetics and shared environment) are of importance for the association studied.

## Results

### Descriptive statistics

Sociodemographic characteristics of the total sample and distributions on the covariates by sexual orientation are presented in Table 1. Sexual minority individuals were younger, had lower income, were more likely to be women, and less likely to be married or cohabitant (all p < .001). There was no significant difference in level of education between the sexual orientation groups. Sexual minority individuals were more likely to receive treatment with antidepressant medications. The proportion of individuals exposure to discrimination and victimization were significantly larger among sexual minority individuals compared to heterosexuals.

**Table 1:** Socio-demographic and covariate distribution among participants in the Swedish Twin by sexual orientation (n = 17,539)

	Sexual minority individuals (n = 1,238)	Heterosexual individuals (n = 16,301)	Sig.
	mean (SD)	mean (SD)	
**Age**	33 (7.7)	34 (7.5)	*P* = < 0.001
**Mean yearly income in SEK** ^**a**^	200.9 (144.4)	223.2 (159.1)	*P* = < 0.001
**Sex**	n (%)	n (%)	*P* = < 0.001
- Women	828 (67)	9667 (59)	
- Men	410 (33)	6634 (41)	
**Relationship status**			
- Married/cohabitant	744 (60)	11,147 (68)	*P* = < 0.001
**Level of education**			*P* = .660
- 12 year or less	530 (45)	7200 (45)	
- More than 12 years	654 (55)	8654 (55)	
**Treatment covariates**			
- Treatment with antidepressant medication	307 (24)	2832 (17)	*P* = < 0.001
**Social stress exposure variables**			
- Discrimination	200 (16)	814 (5)	*P* = < 0.001
- Victimization	56 (5)	145 (1)	*P* = < 0.001

^a^ Swedish kronor, in thousands

### Sexual orientation differences in reduced work ability

The prevalence of SA and DP by sexual orientation group is presented in Table 2. Sexual minority individuals were significantly more likely to have experienced both SA and to be granted DP during the follow-up period, between 2006 and 2018.

**Table 2:** Number of individuals (n) and period prevalence (%) of sickness absence and disability pension granted by the National Social Insurance Agency between 2006–2018 by sexual orientation among Swedish twins

	Sexual minority individuals (n = 1,238)	Heterosexual individuals (n = 16,301)	Sig.
	n (%)	n (%)	
**Sickness absence, 2006–2018**			
- Any diagnosis	333 (27)	3772 (23)	*p* = .003
- Mental diagnosis	104 (8)	955 (6)	*p <* .001
**Disability pension, 2006–2018**			
- Any diagnosis	39 (3)	341 (2)	*p* = .010

Results from logistic regression models showing sexual orientation differences in reduced work ability is reported in Table 3. In the unadjusted models (model 1), sexual minorities were 15% more likely to have experienced a period of sickness absence from work due to any medical diagnosis compared to heterosexuals (odds ratio [OR]: 1.15; 95% confidence interval [95% CI]: 1.05, 1.27). Analyses specifically for mental health SA diagnoses showed that sexual minority individuals were 42% more likely to have experienced a period of SA due to mental health, as compared to heterosexuals (OR: 1.42; 95% 95% CI: 1.17, 1.73). The likelihood for sexual minorities for having been granted DP during the follow-up period was also 58% larger that for heterosexual (OR: 1.58; 95% 95% CI: 1.14, 2.18).

**Table 3:** Odds ratios (OR) with 95% Confidence Intervals (CI) for sexual orientation differences in sickness absence and disability pension granted by the National Social Insurance Agency between 2006–2018 among Swedish twins

	Sickness absence, 2006–2018							
	Model 1 OR (95% CI)^a^	Model 2a OR (95% CI)^b^	Model 2b OR (95% CI)^c^	Model 3 OR (95% CI)^d^	Model 4 OR (95% CI)^e^	Model 5 OR (95% CI)^f^	Model 6 OR (95% CI)^g^	
Any diagnosis								
- Heterosexual individuals	1		1	1	1	1	1	
- Sexual minority individuals	**1.15 (1.05, 1.27)**	1.09 (0.99–1.19)	1.09 (0.99, 1.20)	1.08 (0.98, 1.19)	1.08 (0.99, 1.19)	1.07 (0.97, 1.18)	0.95 (0.76, 1.18)	
Any mental diagnosis								
- Heterosexual individuals	1		1	1	1	1	1	
- Sexual minority individuals	**1.42 (1.17, 1.73)**	**1.32 (1.08–1.60)**	**1.35 (1.10, 1.64)**	**1.33 (1.08, 1.64)**	**1.29 (1.06, 1.57)**	1.18 (0.96, 1.45)	1.11 (0.70, 1.76)	
	**Disability pension, 2006–2018**							
	Model 1	Model 2a	Model 2b	Model 3	Model 4	Model 5	Model 6	
	OR (95% CI)^a^	OR (95% CI)^b^	OR (95% CI)^c^	OR (95% CI)^h^	OR (95% CI)^i^	OR (95% CI)^f^	OR (95% CI)^g^	
Any diagnosis								
- Heterosexual individuals	1		1	1	1	1	1	
- Sexual minority individuals	**1.58 (1.14, 2.18)**	**1.60 (1.16–2.21)**	**1.59 (1.13, 2.23)**	**1.43 (1.02, 2.02)**	1.25 (0.92, 1.71)	-^j^	1.11 (0.53, 2.35)	

^a^ Crude unadjusted model

^b^ Adjusted for age and sex

^c^ Adjusted for all sociodemographic variables (i.e., age, sex, education)

^d^ Adjusted for sociodemographic and social stress exposure (i.e., discrimination and victimization) variables

^e^ Adjusted for sociodemographic variables and treatment with antidepressant medication

^f^ Adjusted for sociodemographic variables, social stress exposure variables, and treatment with antidepressant medication

^g^ Adjusted for familial factors

^h^ Adjusted for social stress exposure variables (model with both sociodemographic variables and social stress variables did not converge)

^i^ Adjusted for treatment with antidepressant medication (model with both sociodemographic variables and antidepressant medication did not converge)

^j^ This model did not converge

### Socioeconomic status and exposure to psychosocial stressors (i.e., discrimination and victimization) as predictors of sexual orientation difference in reduced work ability

The regression models examining the sexual orientation difference in SA from work due to any diagnosis (Table 3), attenuated to non-significant when adjusted for age and sex (model 2; OR: 1.09; 95% 95% CI: 0.99, 1.20). This indicates that the small difference in SA due to any diagnosis between sexual minority individuals and heterosexuals that were observed in unadjusted analyses, was a result of age and sex difference between the two groups. The estimates remained small and non-significant when subsequently adjusted for exposure to psychosocial stressors (model 3).

In the regression models examining the sexual orientation difference in SA due to mental diagnoses (Table 3), the OR was reduced with 17% when the model was adjusted for age and sex but remained significant (model 2; OR: 1.35; 95% 95% CI: 1.10, 1.64). The OR was further slightly reduced in models adjusted for exposure to psychosocial stressors but remained significant (model 3; OR: 1.33; 95% 95% CI: 1.08, 1.64). The reduction in ORs at each of these steps, suggests that both sociodemographic variables and psychosocial stress exposure partially explains the sexual orientation difference in risk of SA due to mental diagnosis.

In the regression models examining the sexual orientation difference in DP due to any diagnosis, the OR remained significant and largely the same when the model was adjusted for age and sex (Table 3, model 2). The OR was reduced slightly in the model adjusted for exposure to psychosocial stressors but remained significant (model 3; OR: 1.43; 95% 95% CI: 1.02, 2.02). This reduction in OR when the model was adjusted for psychosocial stressors, suggests that sexual minorities increased odds of having experienced discrimination and/or victimization partially explained the sexual orientation differences in risk of being granted DP.

### Treatment with antidepressant medication as predictor of sexual orientation difference in reduced work ability

In the next step, regression models were adjusted for treatment with antidepressant medication to estimate how much of the sexual orientation difference in SA and DP that could be explained by increased risk of depression (model 4, Table 3). The results did not change in the model estimating risk of SA for any diagnosis when including treatment with antidepressants, estimates were still non-significant, as was the model adjusted for all covariates (model 5).

The regression model examining the sexual orientation difference in SA due to mental diagnosis showed reduced odds (Table 3, model 4) and in the model adjusted for all covariates including psychosocial stressors (model 5; OR: 1.18; 95% 95% CI: 0.96, 1.45), estimates were non-significant.

In the model examining the sexual orientation difference in DP OR was reduced and non-significant (model 4; OR: 1.25; 95% 95% CI: 0.92, 1.71). The model adjusted for all covariates (model 5) did not converge. The reduction of the ORs in models adjusting for treatment with antidepressant medication indicated that sexual minorities higher likelihood of depression treatment could largely explain the sexual orientation difference in DP.

### Adjustment for familial confounding

The ORs were reduced, and the associations attenuated towards null when the models were adjusted for familial factors, as compared to unadjusted models. The reduction towards null suggest that familial factors had a certain influence on all associations.

## Discussion

Sexual minorities are at greater risk of several health concerns, in particular mental health problems, compared to heterosexuals [28, 29], highlighting the need to understand how this increased risk of poor health is influencing SA and DP. To our knowledge, this is the first study to report on sexual orientation differences in risk of SA and being granted DP in a population-based sample with a co-twin control design. Compared to heterosexuals, sexual minorities were more likely to having experienced SA and having been granted DP during a 12-year follow-up period (2006 to 2018). The odds were highest for DP, where sexual minorities were 58% more likely to having been granted DP compared to heterosexuals. The higher odds for SA due to any diagnosis could largely be explained by age and sex differences. The higher odds of SA due to mental diagnosis could partially be explained by increased risk of being exposed to discrimination and victimization, and partially by having received treatment with antidepressant medication. The higher odds of being granted DP could also partially be explained by increased risk of being exposed to social stress and treatment with antidepressant medication.

In line with research showing that the increased risk of poor health among sexual minorities individuals largely can be explained by exposure to minority stress [1, 16, 30], we found support that exposure to psychosocial stress in the form of discrimination and victimization partially could explain the increased risk of SA and DP. However, further research is needed to increase our knowledge about in what situations psychosocial stress is most detrimental to work ability and what protective and supportive intervention that could buffer against the exposure to such stressors. Research with larger datasets would also enable analyses of potential effect modifiers of the associations identified in the current study and identify specific subgroups, for example based on gender, age, diagnosis, and occupation, that are at particularity high risk of SA or DP. In the current study, we found a reduction of sexual orientation difference in SA in models adjusting for age and sex, indicating that the distribution of these variables by sexual minority status to some degree influenced the increased risk of SA among sexual minorities. This further motivates continued research into the specific sex and age patterns of sexual orientation differences in impaired work ability and its causes.

The reduction of the ORs in models adjusting for treatment with antidepressant medication indicated that sexual minorities higher likelihood of receiving depression treatment at least partially could explain the sexual orientation difference in SA due to mental diagnosis. The increased risk of common mental health problems among sexual minorities has been well documented [11], and it is not surprising that this increased prevalence also increase reduced work ability in terms of SA and DP.

The association between sexual behavior and SA/DP attenuated after familial factors were considered. This could suggest that the associations may also be explained by a common genetic and environmental etiology, but the fact that the estimates for the disparities were non-significant in models adjusted for psychosocial and treatment variables, indicates that genetics and shared - primarily family - environment may have limited influence on sexual minority individuals increased risk of SA and DP. Support for an influence of familial factors of sexual orientation differences has been found in several previous genetically informative studies, where familial confounding was shown in the associations between sexual behavior and psychiatric morbidity [18, 19]. Also, a genetic overlap has been found between sexual orientation and depression, psychoticism, and neuroticism [31, 32]. Although familial confounding regarding SA and DP may seem to be hardly interpretable, the influence of genetic and environmental factors may reflect genetic and/or environmental susceptibility to a disease behind SA and DP. In fact, previous studies have shown genetics to play a role in both SA and DP [33, 34]. Also, SA and DP are complex phenomena, and several non-diseases related, yet heritable, factors have been found to be related to SA and DP, including functional ability [35], birth weight [36], and neuroticism [37].

While this study possesses several notable strengths in sampling (i.e., population-based), assessment of SA and DP (i.e., derived from national registries with no loss to follow-up), and comparison groups (i.e., twins), results must be interpreted in light of several limitations. First, the specific health insurance situation in Sweden likely limits the generalizability of our results, and thus our results are likely unique to this context apart from other countries with similar social security, as for example Finland and Norway. Second, some of the outcomes analyzed in the current study were relatively rare in this sample. The relatively small sample size for having received DP, increases the risk of type 2 error. It is possible that some of the non-significant estimates of sexual orientation differences in treatment for these diagnoses would have been significant with a larger sample, and the results should be interpreted considering this limitation. Third, this study relied on sexual identity as a measure of sexual behavior, precluding examination of whether these findings extend across other dimensions of sexual orientation (e.g., sexual identity, patterns of attraction). Lastly, even with the strong methodology provided by a twin study design, it is not possible to completely account for the complexity of familial influence, given that all family members have a unique experience of that family [38].

In conclusion, this study takes advantage of the high-quality health registry data available in Sweden to conduct the first examination of sexual orientation differences in SA and DP. We found higher period prevalence of both SA and DP among sexual minorities as compared to heterosexuals. The higher odds of SA and DP could partially or fully be explained by sexual orientation differences in sociodemographic factors, exposure to social stress, and treatment for depression. Future studies can extend these findings by continuing to investigate risk factors for SA and DP among sexual minorities and how such factors can be reduced.

## Data Availability

The data that support the findings of this study are available from the original sources: the Swedish Twin Registry, Statistics Sweden, Swedish Social Insurance Agency and the Swedish National Board of Health and Welfare. Restrictions apply to the availability of the data used in this study based on the Swedish Twin project Of Disability pension and Sickness absence (STODS), which were used with ethical permission for the current study and therefore are not publicly available. Data collection and all analyses were performed in accordance with relevant guidelines and regulations. The study was approved after ethical witting by the Regional Ethics Committee in Stockholm (No. 2013/2200-31/2; 2019–06335) and all participants provided their informed consent.

## Code Availability

Code can be made available through contact with corresponding author.
